# Clinical Ethics Committees in Africa: lost in the shadow of RECs/IRBs?

**DOI:** 10.1186/s12910-020-00559-2

**Published:** 2020-11-18

**Authors:** Keymanthri Moodley, Siti Mukaumbya Kabanda, Leza Soldaat, Anita Kleinsmidt, Adetayo Emmanuel Obasa, Sharon Kling

**Affiliations:** 1 Centre for Medical Ethics and Law, Department of Medicine, Faculty of Medicine and Health Sciences, Stellenbosch University, Tygerberg, South Africa; 2 Department of Paediatrics and Child Health, Faculty of Medicine and Health Sciences, Stellenbosch University, Tygerberg, South Africa

**Keywords:** Clinical ethics committees, Clinical ethics consultation service, Africa, Developing countries, Ethics, Clinical ethics, Dilemma

## Abstract

**Background:**

Clinical Ethics Committees (CECs) are well established at healthcare institutions in resource-rich countries. However, there is limited information on established CECs in resource poor countries, especially in Africa. This study aimed to establish baseline data regarding existing formal CECs in Africa to raise awareness of and to encourage the establishment of CECs or Clinical Ethics Consultation Services (CESs) on the continent.

**Methods:**

A descriptive study was undertaken using an online questionnaire via SunSurveys to survey healthcare professionals and bioethicists in Africa. Data were subjected to descriptive analysis and Fischer's exact test was applied to determine associations. Texts from the open-ended questions were thematically analysed.

**Results:**

In total 109 participants from 37 African countries completed the survey in December 2019. A significant association was found between participants’ bioethics qualification or training and involvement in clinical ethics (*p* = 0.005). All participants were familiar with Research Ethics Committees (RECs), and initially conflated RECs with CECs. When CECs were explained in detail, approximately 85.3% reported that they had no formal CECs in their institutions. The constraints to developing CECs included lack of training, limited resources, and lack of awareness of CECs. However, the majority of participants (81.7%) were interested in establishing CECs. Participants listed assistance required in establishing CECs including funding, resources, capacity building and collaboration with other known CECs. The results do not reflect CECs established since the onset of COVID-19 in Africa.

**Conclusions:**

This study provides a first look into CECs in Africa and found very few formal CECs on the continent indicating an urgent need for the establishment of CECs or CESs in Africa. While the majority of healthcare professionals and bioethicists are aware of ethical dilemmas in healthcare, the concept of formal CECs is foreign. This study served to raise awareness of CECs. Research ethics and RECs overshadow CECs in Africa because international funders from the global north support capacity development in research ethics and establish RECs to approve the research they fund in Africa. Raising awareness via educational opportunities, research and conferences about CECs and their role in improving the quality of health care in Africa is sorely needed.

## Background

In medical care, ethical dilemmas abound, adding complexity to decision-making for healthcare professionals (HCPs) [1]. From the HCP’s perspective, resolving such dilemmas is often facilitated by a consultation with a Clinical Ethics Committee (CEC) [1–9]. In addition, CECs have been shown to assist patients, families, or other involved parties to navigate their way out of uncertainty or conflict regarding value-laden concerns that emerge in healthcare [10, 11]. CECs have three main functions in the provision of support services. These include analysis of the dilemmas that arise in the treatment of individual patients, guidance on hospital policy, and education of HCPs [12–16]. Members of CECs are trained to conduct a structured analysis of what is at stake in any given clinical-ethical dilemma, with an emphasis on moral values, moral principles, and relevant law involved in the case [16]. CECs have been widely established in developed countries since the 1970s [17–19]. However, there are limited studies that have assessed CECs in resource poor countries, especially in Africa. This could be attributed to the fact that clinical ethics is not integrated into clinical practice in resource-poor countries [19, 20]. Yet, clinical ethics is a practical discipline that provides a structured approach to assist health professionals in identifying, analysing, and attempting to resolve the ethical problems that arise in clinical practice [21, 22]. Furthermore, in Africa, many people lack adequate resources, access to HCPs, and reasonable healthcare. As a result, focusing on ‘clinical ethics’ is challenging even though there is a strong need for CECs in resource-constrained environments [12, 20]. It is also concerning that, in Africa, ethics deliberation is mainly emphasised in the context of research but not in clinical practice. This is because research ethics is the most developed aspect of bioethics in Africa, with substantial investment and support from international funders from resource rich countries [23, 24]. These funders support clinical research in Africa on local populations but do not include healthcare or clinical ethics in their research awards or capacity development initiatives [24, 25].

In many countries in sub-Saharan Africa such as in Ethiopia [2], Tanzania [23], and Nigeria [25], knowledge of clinical ethics is quite limited among HCPs in the clinical setting [2, 26]. The lack of bioethics training is often a contributing factor to the limited knowledge among HCPs that leaves them unprepared to respond to complex ethical dilemmas. The allocation of scarce resources is also seen as challenging for HCPs who must make difficult ethical decisions [2, 23, 27]. The above-mentioned challenges have also been observed in other developing countries such as Malaysia [28], Nepal [29], and Latin-America [30]. The reality is, with limited knowledge, resources, poor infrastructure, lack of commitment from government and poverty, HCPs find themselves forced to make decisions based on their beliefs or skills, with no ethics support services or committee in place [23, 27].

In South Africa, there are only two reported CECs that assist in dealing with ethical issues [31]. However, since the COVID-19 pandemic, two additional CECs have been established and to our knowledge, there is no documented literature from other parts of Africa. As a result, it is unclear how most African HCPs handle ethical dilemmas in the absence of sufficient ethics expertise. This study aimed to establish baseline data regarding existing formal CECs in Africa to encourage the establishment of CECs or CESs in Africa. Therefore, the objectives of the study were to (1) establish a baseline awareness of existing CECs or CESs in Africa; (2) identify the challenges that exist in countries without formal CECs or clinical ethics services, and (3) identify the kind of assistance needed to establish CECs.

## Methods

### Study design and sampling

A descriptive survey was conducted with 109 HCPs and bioethicists representing 37 African countries during September 2019–December 2019. The sample population was selected based on the existing database of professional networks of HCPs/bioethicists and REC members in Africa that the Centre for Medical Ethics and Law has established over the past 17 years. Through snowball sampling, the study was introduced to other interested parties. Furthermore, the authors also researched and contacted a few delegates from Africa who have presented at the International Conference on Clinical Ethics and Consultation (ICCEC) during previous years.

Ethics approval was granted from the Faculty of Medicine and Health Sciences Health Research Ethics Committee (HREC REF: N19/05/064) at Stellenbosch University, South Africa.

### The survey instrument

The survey tool was developed based on literature [10, 12, 15, 19] and authors’ experience related to clinical ethics in South Africa. A final draft online questionnaire was created using a web-based e-survey, SUNsurveys Checkbox® 6 Version 2018 Q2, and piloted with a group of nine people comprising HCPs and bioethicists. This was to ensure both the validity and reliability of the survey instrument. Thereafter, the online questionnaire was updated to address repetition or incomplete information provided. The updated version of the survey instrument (see Additional file 1) consisted of 16 closed- and 6 open-ended questions. These questions were used to establish baseline data regarding existing formal CECs in Africa. Initially, the survey was developed in English and then translated into French and Portuguese. The latter versions were back-translated to confirm that the translated versions were correct.

### Data analysis

Answers to the online survey were imported into Microsoft Excel for cleaning and thereafter analysed using Statistical Package for Social Sciences (SPSS) version 26. Descriptive statistics were used to calculate response frequencies, percentages, and cross-tabulations. With a low sample size, a Fisher’s exact test with a 0.05 level of significance was used to assess any association between tested variables. There are several possible job positions for HCPs or bioethicists in Africa. Therefore, for this study, job positions were collapsed into the following categories for easy data analysis (see Table 1).

**Table 1:** Demographic characteristics of survey participants (n = 109)

	n (%)
Gender	
Male	75 (68.8)
Female	34 (31.2)
Title	
Prof	32 (29.4)
Dr	52 (47.7)
Mr	10 (9.2)
Mrs	8 (7.3)
Miss	7 (6.4)
*Country	
Uganda	10 (9.2)
Cameroon	7 (6.4)
South Africa	7 (6.4)
Kenya	6 (5.5)
Ethiopia	6 (5.5)
Tanzania	6 (5.5)
Malawi	5 (4.6)
Sudan	5 (4.6)
Rwanda	5 (4.6)
Zambia	4 (3.7)
Egypt	3 (2.8)
Nigeria	3 (2.8)
Zimbabwe	3 (2.8)
Botswana	3 (2.8)
Democratic Republic of the Congo	3 (2.8)
Burkina Faso	3 (2.8)
Gambia	3 (2.8)
Namibia	3 (2.8)
Mozambique	2 (1.8)
Madagascar	2 (1.8)
Mali	2 (1.8)
Type of hospital institution stationed at	
Public	30 (27.5)
Private	8 (7.3)
Doesn't apply (academic institution/non-governmental organization)	71 (65.1)
Position at the institution	
Doctor in academia (*professor, associate professor or researcher*)	40 (36.7)
Doctor in administrative position (chief medical officer and etc.)	11 (10.1)
Doctor in hospital (*general practitioners, senior consultants and *etc.)	10 (9.2)
Lecturer (*in ethics, bioethics or medical sciences*)	18 (16.5)
Administrator (*institutional review board [IRB] administrator, IRB vice-chairman*)	18 (16.5)
Researcher (*medical sciences and bioethics*)	12 (11.0)
Bioethics training or a bioethics qualification?	
Yes	83 (76.1)
No	17 (15.6)
Other training/qualification	9 (8.3)
Type of bioethics qualification	
Certificate	45 (48.4)
Diploma	12 (12.9)
Bachelor	2 (2.2)
Master	20 (21.5)
PhD	7 (7.5)
Postdoc	4 (4.3)
Other	3 (3.2)

^*^Other countries with one respondent: Algeria, Mauritius, Swaziland, Sierra Leone, Guinea, Eritrea, Togo, Senegal, Libya, Seychelles, Angola, Morocco, and Tunisia

Using qualitative thematic analysis, responses from open-ended questions were analysed, which involved extensive familiarisation with the data by reading and identifying recurring responses [32].

## Results

### Demographic information

A total of 155 people were invited to the research study and 109 completed the online survey, yielding an overall response rate of 70.3%. The respondents were from 37 different countries, but 13 countries had only one respondent (Table 1). Almost half of the participants (47.7%) were either a medical doctor or PhD graduate with a male predominance (68.8%). Most of the participants (65.1%) were attached to an academic institution/non-governmental organization while 27.95% were based at public hospitals.

Regarding their position at the institution, almost 37% of the participants were doctors in academia and consulted occasionally at the hospitals/clinics. This was followed by lecturers and administrators (16.5% each, respectively). Most participants had bioethics training (76.1%) with 48.4% possessing a certificate in bioethics and 21.5% a master’s degree. There were a few respondents with a doctoral degree in bioethics (7.5%).

### Description of clinical ethics committees

Most of the participants (72.5%) indicated that they were involved in clinical ethics, bioethics organizations, or institutions (Table 2) while 27.5% mentioned that they were not involved in clinical ethics or bioethics but either had training or a qualification in bioethics. A significant association was found between participants’ bioethics qualification or training and being involved in clinical/medical ethics (*p* = 0.005). However, no significant association was found between gender, a position at the institution, type of hospital institution, and involvement or awareness of CECs.

**Table 2:** Description of clinical ethics committees (n = 109)

	n (%)
Involved in any clinical ethics/medical ethics/bioethics organisations or institutions?	
Yes	79 (72.5)
No	30 (27.5)
Do you have an established clinical ethics committee/healthcare ethics service at your institution/organisation?	
Yes	16 (14.7)
No	93 (85.3)
*Members on the committee?	
0–5	1 (6.3)
6–10	9 (56.3)
11–15	5 (31.3)
16–20	1 (6.3)
*Disciplines represented by CEC members (more than one option to choose)	
Law	9 (60)
Bioethics	8 (53.3)
Social sciences	7 (46.7)
Health sciences	13 (86.7)
Community	3 (20)
Religious	6 (40)
Management	6 (40)
*Frequency of the committee having scheduled meetings	
Weekly	1 (6.3)
Monthly	6 (37.5)
Quarterly	7 (43.8)
Annually	0
Other	2 (12.5)
*Frequency of the committee meeting for ad hoc consultations (when an urgent dilemma arises)	
Less than 10 times a year	12 (75)
Between 10 and 20 times a year	3 (18.8)
Between 21 and 30 times a year	0
Other	1 (6.3)
*Typical problems referred to the committee (more than one option to choose)	
Treatment declined (including Jehovah’s witnesses)	6 (37.5)
Withdrawal of life support	5 (31.3)
Termination of pregnancy	4 (25)
Consent	11 (68.8)
Paediatrics	6 (37.5)
HIV related	5 (31.3)
Social media use	1 (6.3)
Innovative treatment	8 (50)
Traditional treatment	4 (25)
Other	5 (31.3)
Constraints to developing a CEC (more than one option to choose)	
Limited resources	31 (36.9)
Understaffed	22 (26.2)
Lack of training	46 (54.8)
Other	40 (47.6)

^*^Represent the number of participants that indicated that they have CECs

All participants were familiar with Research Ethics Committees (RECs), and initially conflated RECs with CECs. When CECs were explained in detail, approximately 85.3% reported that they had no formal CECs in their institutions, organizations or countries. This finding suggests that formal CECs have not been established in most African countries.

The 14.7% of participants who mentioned that they had CECs in some of the hospitals or institutions were from the following countries: Egypt, Kenya, Ethiopia, Cameroon, Algeria, South Africa, the Democratic Republic of Congo, Gambia, Eritrea, Sudan, and Senegal. From these country representatives, most of the total number of committee members (56.3%) ranged from six to ten. Most CEC members included people from disciplines in health sciences (86.7%), law (60%), and social sciences (46.7%) with community or lay members accounting for 20% of CECs membership. Approximately 44% of the committees mentioned that they met quarterly for their scheduled meetings, whereas 37.5% met monthly. Seventy-five percent of the CECs met less than 10 times a year for ad hoc consultations, while 18.8% reported that they met between 10 and 20 times a year.

When asked about ethical dilemmas that were presented at the committee, most indicated issues of consent (68.8%), declined treatment (including Jehovah's witnesses) (37.5%), and ethical issues related to children (37.5%).

Participants who indicated that they lacked CECs in their institution, mentioned that lack of training (54.8%) was a prominent reason. Other reasons included lack of interest in CECs, lack of knowledge of the importance of CECs, lack of time to establish CECs, and limited resources (36.9%) as the common constraints to developing a CEC. These reasons are reflected in the quotes below:

Some medical doctors don’t understand the importance of medical ethics, also issue of logistics regarding establishing committees [Country 16]

Lack of knowledge and training. I doubt if most the clinicians in our setting have heard of such a committee before. So the first step to help us establish a clinical ethics committee would be to sensitize clinicians about it's need, train people who would be members and administrators of the committee and may be later ensuring its sustainability by helping in its integration with the other clinical services [Country 19]

Non-prioritisation by health institutions [Country 13]

I think it is about the existing system in the university which only knows research ethics committees. The awareness of the leaders of the college and even the clinicians are not as such having experience in this regard. It might need awareness creation and training on the importance of the clinical ethics committee. [Country 19]

### Other CECs or consultation ethics services

Most participants indicated they were not aware of other CECs or CESs in their institution (62.4%) or country (60.6%) (Table 3). They mentioned reasons for not having these services that were similar to the constraints of developing CECs.

**Table 3:** Other CECs or consultation ethics services (N = 109)

	n (%)	
	Yes	No
Other services like cess that assist in ethical decision-making At your institution	41 (37.6)	68 (62.4)
Aware of any other CECS or similar services in your country	43 (39.4)	66 (60.6)
Interested in establishing a CEC at your institution	89 (81.7)	20 (18.3)

Other reasons were that their institutions were primarily research-based, while some had no idea why such services did not exist. As for those participants who indicated there were other similar services to clinical ethics consultation, most commented on services related to reviewing research protocols and clinical trials as illustrated in the comments below:

We have a research ethics committee at our institute for reviewing all protocols [Country 16]

We have a Clinical Research Approval Committee (Chief Clinical Officer, Legal Executive and Nursing Executive) to co-ordinate research activities in the hospital [Country 20]

The service is based at the University teaching hospital to assist and solve some ethical issues regarding the standards of care, but they don't meet regularly, and also tend to create confusion between research ethics and clinical ethics [Country 21]

Interestingly, most participants (81.7%) were interested in establishing a CEC at their institution. They mentioned the kind of assistance that they would require in establishing a CEC which included: training or mentorship on CECs and their membership, financial assistance or funding in setting up CECs, resources, capacity building, and collaboration with other known CECs:

We would require finances for having the members trained on clinical ethics so that they offer good services while in the clinical ethics consultation [Country 7]

I will need further training in clinical ethics and perhaps a benchmarking exercise to see exactly how these operate and guidance in developing policies, guidelines and tools that govern their operations [17]

Lots of deficiencies with regards to trained personnel and organizational capacity. I believe short-term trainings and institutional capacity in terms of guideline development would strengthen and revitalize CECs. [Country 19]

I am a trained clinical ethicist in addition to being a masters level educated bioethicist… l just need a mix of the right professionals who are willing and interested to start a CEC. Where indicated l have CEC professional colleagues outside my country who are willing to come as external resource persons if needed. A problem l encountered in my country was a lack of interest of medical clinicians in ethical issues. Clinical dilemmas of an ethical nature were seen as a medical model problem to resolve, most usually single handedly, by the medical doctor, in a paternalistic manner [Country 2]

## Discussion

To our knowledge, this is the first study to examine the presence or existence of established CECs in Africa. The respondents hailed from 37 different countries on the continent. The findings reported in this paper focused on the responses to questions related to the presence of CECs in hospitals or institutions, the role of CECs, and assistance in establishing CECs in Africa. Most of the respondents had bioethics training with the majority possessing a certificate in bioethics. In addition, our findings showed a significant association between bioethics qualification or training and being involved in clinical ethics. This association suggests that professionals with bioethics qualifications or training are more likely to be involved in or contribute to clinical ethics. This finding was corroborated in a study in Germany where a correlation between ethics training or qualifications and the success of ethics services or clinical ethics committees was found [33]. It is important to note, however, that having an ethics qualification or training is not necessarily an indication of knowledge or awareness of clinical ethics. If the original bioethics training focused on research ethics, as many capacity development programs funded by the European and Developing Countries Clinical Trials Partnership (EDCTP) and the National Institutes of Health (NIH) do, then clinical ethics is usually not part of the training curriculum.

Only 14.7% of participants mentioned that they had established CECs in their countries. However, it transpired after cleaning the data and confirming with the participants whether they were referring to CECs or RECs, that most of the respondents were actually referring to RECs. This was because they had no knowledge of CECs. Similar findings have been reported in Latin-America [30]. The American Academy of Pediatrics, in their Policy Statement on institutional ethics committees, clearly distinguishes between RECs and CECs [34].

Research ethics is typically common and established in most African countries [19, 24] as there is abundant funding from the United States (NIH) and Europe (EDCTP) to establish RECs so that clinical trials funded by these organisations may be reviewed in Africa. Healthcare and clinical ethics are not prioritised by funders whose interest lies more with research than healthcare despite the two disciplines intersecting inextricably in multiple ways. It may be argued that a disproportionate amount of funding is allocated to establishing RECs in Africa and that at least some of that funding ought to be diverted to clinical ethics. Likewise training curricula ought to include bioethics more broadly and not just research ethics.

When participants were questioned about whether they had established CECs or CESs in their institution, the majority (85.3%) had not. This could be because there are limited resources to establish CECs in developing countries, including Africa, where clinical ethics is not a priority [19, 20, 23]. Africa bears a double burden of infectious and non-infectious diseases, thus posing a challenge to already weak healthcare systems. In addition, the high patient load, poor infrastructure, and lack of resources, including insufficient numbers of qualified health workers, make it difficult to focus on clinical ethics, despite it being regarded as necessary and important [2, 19, 23]. Preliminary findings from our study suggest a strong need to establish CECs in Africa. In Tanzania, the key point that emerged from the Dartmouth/Penn Research Ethics Training and Program Development for Tanzania workshop was the prioritization and recognition of the need for the establishment of CECs or CESs [23]. Similarly, the need for CESs has also been emphasised in another study conducted in Gabon [35].

Our data also showed that CEC composition included diverse membership with representation from disciplines such as health sciences (86.7%), law (60%), and social sciences (46.7%). Interestingly, community or lay members (20%) were also present. Studies in resource-rich countries have highlighted the need for community members, as they provide a different specific and irreducible perspective, which will contribute to better participation, transparency, diversity and fairness in decision-making [36, 37].

South Africa has two established CECs: at Tygerberg Hospital in Cape Town and Grey’s Hospital in Pietermaritzburg [31]. Ethical issues presented to the CEC at Tygerberg Hospital include withholding and/or withdrawing life-sustaining treatment and issues regarding consent, including substitute decision-making. Interestingly, consent was also regarded as the most commonly reported ethical dilemma to the CECs in our study. Although the presence of CECs or consultation services can be helpful for HCPs, there are constraints in developing these committees within the African context. Most of the participants indicated a lack of training and other constraints such as HCPs with poor knowledge or who do not understand the importance of CECs. For example, in a study conducted in Nigeria, it was found that there were gaps in the knowledge and practice of healthcare ethics among HCPs [26]. The study found 68.7% of respondents agreed that “the doctor should do what is best” irrespective of the patient’s wishes [26]. The lack of knowledge in clinical ethics has also been observed in an Ethiopian study that assessed the clinical ethics dilemmas faced by physicians working in public hospitals. One of the dilemmas concerned observed unethical or inappropriate care, and this was because the physicians lacked the skills for the procedures, did not know how to carry out the procedure or because no one had more advanced training [2]. These findings suggest the urgent need for educational interventions among bioethicists and HCPs in Africa to create awareness and improve decisions when deliberating on clinical ethics dilemmas.

Other study participants mentioned a lack of interest in CECs or time to establish CECs. It is important for health professionals or bioethicists in Africa to establish the interest of professionals and to encourage interested individuals to pursue training in clinical ethics [20]. In a systematic review conducted by Ong et al. [1], it was found that most training programmes failed to provide CEC trainees with enough knowledge, skills, and experience to meet required competencies. Furthermore, many CEC training programmes were not supported by host institutions which affected the sustainability of the programmes and long-term support of CEC trainees [1]. Therefore, it is important when such training programmes are established in Africa that they are supported by institutions to ensure uniformity and sustainability of training for their members to deliver their services effectively. Another constraint mentioned was limited resources, which are also among the ethical challenges faced in South African healthcare [31]. Similarly, in a Tanzanian study, participants mentioned that they had few or no resources to assist them to resolve ethical issues when these arose [23]. There was a similar observation in an Ethiopian study, where resource scarcity influenced the physicians in numerous ways and forced them into ethically challenging situations [2]. This underscores the need for resources to establish CECs in Africa. The general lack of resources and poor awareness of CECs in African healthcare settings gives rise to clinical ethics dilemmas which would be best resolved by CECs. Paradoxically, however, the lack of resources also mitigates against the training of staff in clinical ethics and the development of CECs.

The majority of the participants mentioned that they were interested in establishing CECs in their institutions and countries. This indicates that the HCPs and bioethicists are cognisant of the need for CECs in health care. The participants in our study also indicated the kind of assistance which has previously been discussed, including financial assistance or funding in setting up CECs, capacity building and collaboration with other known CECs. For example, for a CEC to be established members will require ethics education and this will need additional financial support for training [23].

There is an urgent need for implementing clinical ethics education within medical schools to raise students’ awareness and to enable HCPs to tackle ethical dilemmas in their daily practice. In 1999, the World Medical Association recommended that medical ethics should be included in all medical school curricula, including as a continuing professional development requirement [38]. Our findings suggest that a two-tiered approach might work well to encourage the establishment of CECs in Africa. While bioethicists are best placed to create interest in and to set up CECs, at the same time, health sciences education would benefit from bioethics training programs that include clinical ethics and research ethics. These two strategies could work synergistically, and the natural consequence would be a heightened awareness of clinical ethics and the establishment of CECs and/or CESs. We recommend that more studies are done to assess how CECs can be established in a sustainable manner in Africa.

### Study limitations

There are some limitations that must be taken into account when interpreting the results of this study. There was a predominance of participants from countries such as Uganda, South Africa, Cameroon, etc. in comparison with other countries. The reason for this could be that it was easier to obtain active email contacts from these countries. We aimed to get a minimum of one response per African country to include at least one representative from each country. In addition, it is possible that we could have missed HCPs or bioethicists that work within healthcare institutions or organizations that have CECs. Despite these limitations, this study demonstrates the need for future research on the use of established CECs among HCPs in African countries.

This study also investigated more detailed information using qualitative methodology research on awareness and perception of the participants regarding CECs in Africa. These findings will be published in a separate paper.

## Conclusion

This study has provided us with a snapshot of CECs in the African context, where there are very few CECs. While HCPs and bioethicists were familiar with RECs, they had a low level of awareness/knowledge of CECs. Research ethics and RECs overshadow CECs in Africa because international funders from the global north support capacity development in research ethics and establish RECs to approve the research they fund in Africa. Expanding such funding opportunities to CECs is critical. A two-tiered approach to facilitate the establishment of CECs in Africa is recommended. This would involve the encouragement of bioethicists in Africa to stimulate CEC development in their institutions (via conferences and networks) while simultaneously increasing clinical ethics content in bioethics curricula at undergraduate and postgraduate levels in health sciences education. The coronavirus pandemic has brought into sharp focus the pivotal role that CECs can play in hospitals. This may provide the impetus needed to create CECs in Africa.

## Supplementary information

**Additional file 1.** Survey instrument.

## Data Availability

Anonymised datasets used during the current study are available from the corresponding author.

## Abbreviations

CEC: Clinical Ethics Committee
HCPs: Healthcare Professionals
REC: Research Ethics Committee
SPSS: Statistical Package for Social Sciences
CES: Clinical Ethics Consultation Service
IRB: Institutional Review Board
