# Supplementary material for: Clinical Ethics Committees in Africa: lost in the shadow of RECs/IRBs?
Source: BMC Med Ethics. 2020 Nov 18;21:115. doi: 10.1186/s12910-020-00559-2 (PMC7672173; doi:10.1186/s12910-020-00559-2)
Supplement: Supplementary file 1 — Additional file 1. Survey instrument. [file 12910_2020_559_MOESM1_ESM.docx]

**Promoting the establishment of Clinical Ethics Committees in Africa**

Healthcare in the 21st century is fraught with ethical dilemmas. With the increasing awareness surrounding the ethical dimensions of clinical practice, Clinical Ethics Committees (CECs) are established to provide services that ensure high standards of ethical practice. CECs are crucial in clinical settings.

Most healthcare workers are aware of research ethics, but not as familiar with clinical ethics. There are a multitude of ethical dilemmas arising in the clinical setting. These dilemmas might involve difficulties around prioritisation of care, resource allocation, treatment without consent, disclosure of health status status to partners or parents, termination of pregnancy, patients’ refusal of treatment, etc.

This study is being conducted to encourage the establishment of CECs in Africa and to ascertain where CECs are located in Africa.

Participants' feedback from the survey will be kept confidential. Please understand that your participation is voluntary. You may decline to participate or you can withdraw from participation any time you wish with no consequences. However, I would be grateful if you would assist to answer the full questionnaire.

The survey will take you approximately 10 minutes.

For further information or inquiries, please contact the administrator at bioethics@sun.ac.za

***I confirm that I have read and understood the information provided on this study**

- Yes
- No

***I agree to take part in this survey and that the data generated will be used for research purposes**

- Yes
- No

**Gender**

**Title**

- Prof
- Dr
- Mr
- Mrs
- Miss

***Country**

***Type of hospital institution stationed at**

- Public
- Private
- Doesn’t apply

**What is your position at the institution?**

***Do you have bioethics training or qualification?**

- Yes
- No
- Other training/qualification

If yes, please indicate the bioethics qualification. Tick all that apply

- Certificate
- Diploma
- Bachelor's Degree
- Masters Degree
- PhD
- Postdoctoral
- Other:

***Are you involved in any clinical ethics / medical ethics / bioethics organisations or institutions?**

- Yes
- No

***Do you have an established clinical ethics committee / healthcare ethics service at your institution/organisation?**

- Yes
- No

**In your opinion, what are the constraints to developing a CEC?**

- Limited resources
- Understaffed
- Lack of training
- Other:

***How many members are on the committee?**

- 0-5
- 6-10
- 11-15
- 16-20

**What disciplines do the CEC members represent? Tick all that apply**

- Law
- Bioethics
- Social Sciences
- Health sciences
- Community
- Religious
- Management
- Other:

**How often does the committee have scheduled meetings?**

- Weekly
- Monthly
- Quarterly
- Annually
- Other:

**How often does the committee meet for ad hoc consultations? (when an urgent dilemma arises)**

- Less than 10 times a year
- Between 10 and 20 times a year
- Between 21 and 30 times a year
- Other:

***What are typical problems referred to your committee? Tick all that apply**

- Treatment declined (Including Jehovah’s witnesses)
- Withdrawal of life support
- Termination of pregnancy
- Consent
- Pediatrics
- HIV related
- Social media use
- Innovative treatment
- Traditional treatment
- Other:

***Are there services similar to clinical ethics consultation services, to assist in ethical decision-making at your institution?**

- Yes
- No

*If yes please describe the service

If no, what are the reasons for not having these services?

**Do you know of any other CECs or similar services in your country?**

- Yes
- No

**Would you be interested in establishing a CEC at your institution?**

- Yes
- No

If yes, what assistance would you require in order to do so?

**Would you be interested in attending a conference on establishing CECs in Africa if it is held on the continent?**

- Yes
- No

**Thank you for participating in the study**
